# Long-term storage of feces at −80 °C versus −20 °C is negligible for 16S rRNA amplicon profiling of the equine bacterial microbiome

**DOI:** 10.7717/peerj.10837

**Published:** 2021-03-09

**Authors:** Stefan Gavriliuc, Mason R. Stothart, Astrid Henry, Jocelyn Poissant

**Affiliations:** Faculty of Veterinary Medicine, University of Calgary, Calgary, AB, Canada

**Keywords:** Microbiome, 16S, DNA metabarcoding, Long-term storage, *Equus ferus* caballus, Protocol, Sequencing, Horse, Amplicon, Equine

## Abstract

The development of next-generation sequencing technologies has spurred a surge of research on bacterial microbiome diversity and function. But despite the rapid growth of the field, many uncertainties remain regarding the impact of differing methodologies on downstream results. Sample storage temperature is conventionally thought to be among the most important factors for ensuring reproducibility across marker gene studies, but to date much of the research on this topic has focused on short-term storage in the context of clinical applications. Consequently, it has remained unclear if storage at −80 °C, widely viewed as the gold standard for long-term archival of feces, is truly required for maintaining sample integrity in amplicon-based studies. A better understanding of the impacts of long-term storage conditions is important given the substantial cost and limited availability of ultra-low temperature freezers. To this end, we compared bacterial microbiome profiles inferred from 16S V3–V4 amplicon sequencing for paired fecal samples obtained from a feral horse population from Sable Island, Nova Scotia, Canada, stored at either −80 °C or −20 °C for 4 years. We found that storage temperature did not significantly affect alpha diversity measures, including amplicon sequence variant (ASV) richness and evenness, and abundance of rare sequence variants, nor presence/absence, relative abundances and phylogenetic diversity weighted measures of beta diversity. These results indicate that storage of equine feces at −20 °C for periods ranging from a few months to a few years is equivalent to storage at −80 °C for amplicon-based microbiome studies, adding to accumulating evidence indicating that standard domestic freezers are both economical and effective for microbiome research.

## Introduction

Targeted marker gene sequencing allows for the characterization of biological communities at high-resolution (Cristescu, 2014). In particular, quantifying the resident bacteria of the gastrointestinal tract, or the gut microbiome, using deep sequencing of 16S rRNA amplicons has furthered our understanding of host physiology and disease etiology, and improved clinical diagnostics and treatment of gut dysbiosis (Kim et al., 2017; Vandeputte et al., 2017). As the cost of Next-Generation Sequencing (NGS) experiments continues to fall, marker gene surveys have become increasingly commonplace. However, the findings of different studies are often not readily comparable or generalizable because of a lack of standardized methodology (Vandeputte et al., 2017). For example, the use of different DNA extraction kits and amplification methodologies have been observed to introduce technical variation comparable to that of biological variation in metagenomic and amplicon-based experiments (Costea et al., 2017; Sinha et al., 2017). While these effects are substantial and well-documented, the spread of marker gene surveys has brought with it a renewed interest in optimizing and finding alternative sample storage solutions, which are also known as large sources of technical variation (Kim et al., 2017).

Short-term processing and storage of fecal samples for microbiome research has received considerable attention due to their importance in clinical applications (Table 1). Where immediate extraction is not possible, the conventionally accepted gold standard for short-term storage is rapid freezing at −80 °C without buffer (Vandeputte et al., 2017). While the process of freezing samples is still under contention (Bahl, Bergström & Licht, 2012; Fouhy et al., 2015; Metzler-Zebeli et al., 2016), it remains by far the most common approach for preventing rapid changes in the abundance of specific taxa under ambient conditions following sampling. This phenomenon, often referred to as microbial ‘blooms’, has been well documented. For example, a meta-analysis has shown that human rectal swabs stored at room temperature experience characteristic growths of *Gammaproteobacteria* (Amir et al., 2017). A similar increase in the abundance of *proteobacteria* was also seen in the microbiota of tadpoles (*Nanorana parker*) gut samples left thawing at ambient temperature for 12 h (Anslan et al., 2019), while equine feces collected 12 h post-defecation showed sharp increases of families from the *Firmicutes* phylum (Beckers, Schulz & Childers, 2017). Though the effect of sample storage at ambient temperature has been well-documented across study systems, it has remained unclear whether freezing at −80 °C is overly conservative. Notably, an increasing number of studies have claimed or demonstrated that storage at −20 °C is generally sufficient over the short-term (Song et al., 2016; Blekhman et al., 2016; Panek et al., 2018; Wang et al., 2018). Overall, the consensus emerging from these studies is that (any) differences in microbiome profiles caused by variation in freezing temperature are typically negligible when compared to biological patterns of interest (Lauber et al., 2010; Carroll et al., 2012; Dominianni et al., 2014; Blekhman et al., 2016; Bundgaard-Nielsen, Hagstrøm & Sørensen, 2018) and that storage at −80 °C may therefore be unnecessary for storage periods less than a few weeks or months.

**Table 1:** Summary of studies having investigated the effect of freezing storage temperature (−20 °C or −80 °C) on microbiome profiling using 16S amplicon sequencing. Selected studies have compared samples stored at different freezing temperature without buffer. Studies are ordered by storage duration.

Duration	Temp (°C)	Control	Sample	16S Region	Diversity measure (statistical test/method)	Conclusions	References
~1 day	−20 °C + 1–2 freeze thaw cycles, then -80 °C	−80 °C	Human stool and rectal swab	V4	α: Richness (Kruskal–Wallis) β: Yue & Clayton dissimilarity index (PCoA, Wilcoxon)	α: No significant effect β: Samples clustered by host (not storage method), no difference in θ_YC_ between storage temperatures	Bassis et al. (2017)
1 or 3 days	-20°C	−80 °C	Human stool	V4	α: Richness, Shannon (ANOVA & Tukey post-hoc/Kruskal-Wallis & Dunn’s post-hoc) β: Hellinger distances (PCA) OTU – 25 most abundant taxa	α: No significant effect β: Samples clustered by host rather than storage method OTU: No significant effect	Bundgaard-Nielsen, Hagstrøm & Sørensen (2018)
1 week	−20 °C	−80 °C	Human stool	V1–V3	α: Richness, Chao1, Shannon, Phylogenetic Diversity whole tree (Wilcoxon signed rank) β: Weighted & unweighted UniFrac, Bray-Curtis (UPGMA & PCoA clustering, Mann–Whitney *U* test, PERMANOVA) OTU: Pairwise comparisons of specific taxa (*G*-test)	α: No significant effect except for Shannon diversity in one sample β: Samples clustered by host rather than storage temperature; no significant difference between temperatures OTU: No effect	Tedjo et al. (2015)
3 & 14 days	−20 °C	-80°C	Human stool & skin, soil	V1–V2	α: Faith’s phylogenetic diversity (Kruskal–Wallis) β: UniFrac (PERMANOVA, NMDS) OTU: Relative taxon abundance (Kruskal–Wallis)	α: No effect β: No effect OTU: minor differences in abundance of specific taxa but no significant effect on overall community composition	Lauber et al. (2010)⁠
4 weeks	−20 °C, −80 °C	Fresh	Human pharyngeal	V1–V3	α: Richness (Dirichlet multinomial models) β: Weighted UniFrac (PCoA, Dirichlet multinomial models) OTU: Relative taxon abundance (metagenomeSeq)	α: No significant effect β: No significant effect OTU – No significant effect	Hang et al. (2014)
33 days	−20 °C, −80 °C	Fresh	Murine stool	V3–V4	α: Richness, Chao1(two-tailed Student’s *t*-test, non-parameteric Mann–Whitney) β: Bray-Curtis (PCoA)	α: No significant effect β: Samples clustered by host rather than storage temperature)	Jenkins et al. (2018)
5 weeks total	−20 °C (1 week) then −80 °C (4 weeks)	Fresh	Human mid-vaginal swab	V1–V2	β: Jensen-Shannon divergence, relative entropy, Euclidean distance, Bray–Curtis (Kolmorgorov–Smirnov)	β: No difference between storage temperatures	Bai et al. (2012)
Up to 8 weeks	−20 °C, −80 °C	Fresh	Spider monkey stool	V4	α: Shannon, Simpson (LSMEANS) β: Weighted & unweighted UniFrac (PERMANOVA, PCoA, distance boxplot analysis) OTU: Mean frequencies (Kruskal–Wallis, decision trees)	α: No significant effect β: Fresh samples clustered with frozen samples, frozen sample not significantly different from fresh samples OTU: Frozen samples misassigned to each other	Hale et al. (2015)
3 months	−20 °C, −80 °C	Fresh	Porcine fecal samples	Taxa specific	OTU: Relative and absolute abundances of specific groups (linear discriminant analysis)	OTU: Frozen samples most similar to each other	Metzler-Zebeli et al. (2016)
14 years	Freeze-dried then −20 °C	Matched cohort stored at −80 °C	Human stool	V3–V4	β: Bray–Curtis, weighted & unweighted UniFrac (ANOSIM, NMDS)	β: Significant difference in Bray–Curtis dissimilarities but not UniFrac distances, clustering overlap between −20 °C and matched cohort	Kia et al. (2016)

While the impacts of short-term storage temperature on microbiome profiles are relatively well understood, less is known of the impacts of storage conditions over long periods of time. Delineating short-term from long-term storage is necessarily subjective, but a gap exists in the literature for storage periods greater than a few weeks or months (Table 1). A notable exception is the study of Kia et al. (2016) which showed that the microbiota from freeze-dried human fecal samples stored for 14 years at −20 °C were similar to those of samples stored at −80 °C in a cohort matched for age, sex, BMI and pre-existing health conditions (McDonald et al., 2018)⁠. However, a significant difference was nonetheless observed between groups when considering Bray-Curtis dissimilarities, and it is unclear if similar results would be obtained without the use of freeze-drying which is an uncommon practice in microbiome studies.

In the absence of relevant research, −80 °C has been widely adopted as the gold standard for long-term storage of biological samples intended for microbiome analyses including feces (Hale et al., 2015; Jenkins et al., 2018; Shaw et al., 2016), necessitating the use of ultra-low temperature freezers. Compared to regular −20 °C freezers, ultra-low temperature freezers incur higher capital and energy costs, have higher carbon footprints, and lower durability (Gumapas & Simons, 2013). In addition to constraints imposed by those with limited funding and resources (Vogtmann et al., 2017; Carruthers et al., 2019), immediate or prompt transfer to −80 °C freezers is also often logistically impractical, especially for studies conducted in remote areas (Song et al., 2016; Blekhman et al., 2016). Ultimately, meeting current gold standards often impose significant financial and logistical burdens on researchers, and failure to meet these standards creates uncertainty regarding sample integrity and the validity and comparability of results.

Given the need to validate storage protocols currently in use by our group and others for projects where access to −80 °C storage is limited or impossible, we evaluated the effect of long-term freezing storage temperature on fecal samples collected as part of a long-term study of feral horses on Sable Island, Nova Scotia, Canada (see Gold et al., 2019 and Regan et al., 2020 for details about this study). Specifically, we tested whether storing aliquots from the same fecal sample at either −20 °C and −80 °C for 4 years affected 16S rRNA amplicon characterization of the equine bacterial microbiome. This study is among the firsts to explicitly test the for impacts of long-term freezing temperature on 16S microbiome profiling, and will contribute to protocol development and evaluation in microbiome research.

## Materials and Methods

### Field samples

The fecal samples used in this study were obtained from the population of feral horses on Sable Island, Nova Scotia, Canada (43°55′N; −60°00′W). We collected the surface portion (not in contact with the ground) of eight freshly voided fecal samples using nitrile gloves which were then inverted and sealed. Samples were kept on ice in the field for a maximum of 7 h before being sub-sampled into 1−2 mL cryovials on the same day. Paired samples (aliquots) were then either stored immediately in a liquid nitrogen dewar and subsequently transferred to a −80 °C freezer or stored immediately in −20 °C chest freezers for 4 years. Sample collection and subsequent laboratory analyses were performed under Parks Canada Agency Research and Collections Permit SINP-2013-2014, University of Saskatchewan Animal Care Protocol 20090032, and University of Calgary Animal Care Protocol AC18-0078.

### DNA extraction and sequencing

We extracted DNA from 0.2 g sub-samples of feces using a single Qiagen Powersoil kit (Qiagen, Hilden, Germany) following the manufacturer default recommendations. The protocol followed Qiagen’s Kit Handbook with homogenization performed at full speed for 10 min using the 2 mL bead beating tubes provided with the kit (0.7 mm Dry Garnet) and a Vortex-Genie 2 fitted with Qiagen’s Vortex Adapter (Cat. No. 13000-V1-24). All samples were vortexed simultaneously with paired samples distributed randomly across the Vortex Adapter. DNA extracts were quantified using the Qubit dsDNA BR Assay Kit and standardized to 20 ng/uL with molecular grade water prior to polymerase chain reaction (PCR) amplification. PCR and sequencing was outsourced to the University of Calgary Centre for Health Genomics and Informatics. Library preparation, including PCR program and conditions, was done as specified in the Illumina 16S Metagenomic Sequencing Library Preparation protocol Part # 15044223 Rev. B. Briefly, PCR was conducted on the V3–V4 region of the 16S rRNA gene using the 341F/805R Primers. Sequencing was conducted on an Illumina MiSeq platform using v3 chemistry (600-cycle) to generate 2 × 300 base pair (bp) paired-end reads using dual 8 bp indexing, a loading concentration of 4 pM and 25% phiX spike-ins. Lastly, we used 2 blanks containing only water and a no-template control (NTC) as negative controls to assess for contamination from extraction and sequencing.

### Bioinformatics

Raw reads were demultiplexed and indices removed during the MiSeq post-run processing. Cutadapt v2.8 (Martin, 2011) was used to remove sequencing primers in paired-end mode with the number of searches for a primer in a sequence raised to *n* = 5. Sequences with ambiguous nucleotides were removed using the *filterAndTrim* function from the DADA2 package (Callahan et al., 2016). DADA2 v1.16 with the corresponding tutorial was used to process primerless sequences and extract amplicon sequence variants (ASVs). Quality filtering was conducted with truncation lengths set to 250 and 200 for forward and reverse reads, respectively, while the maximum number of expected errors were left unchanged. The number of rounds to convergence was increased from 10 to 15 for constructing empirical sequencing error models. ASVs were extracted using the core *dada* algorithm with the option for pooling sequences set to TRUE. Forward and reverse reads were subsequently merged using default parameters with *mergePairs*, and chimeras were removed using the *consensus* method. ASVs represented by a single copy in the final ASV table (singletons) were excluded.

Taxonomy was assigned using the DADA2 implementation of the RDP classifier algorithm, *assignTaxonomy*, with the Silva 138 reference taxonomy database and an 80% confidence threshold. For phylogenetic-based beta diversity analyses, we aligned all ASVs using *AlignSeqs* from the DECIPHER package v2.16.1 (Wright, 2015) and created a neighbor-joining tree using the *NJ* command from the *phangorn* package v2.5.5 (Schliep, 2011) using default parameters. Finally, we used the decontam v1.8.0 package (Davis et al., 2018) to remove contaminant ASVs using the prevalence method and the default threshold for detection.

### Statistics

Statistical analysis was conducted with the *vegan* v2.5-6 (Oksanen et al., 2019) and *phyloseq* v1.32.0 (McMurdie & Holmes, 2013) packages unless otherwise noted. Prior to statistical analysis, samples were rarefied to the minimum sequencing depth (45,832) among all samples using *rarefy_even_depth()*. We quantified alpha diversity using observed richness, Shannon and Chao1 diversity indices for rarefied ASV counts. Observed richness and the Chao1 index were normally distributed and compared between temperature treatments using paired two-sided *t*-tests, while Shannon indices were non-normally distributed and therefore compared using a Wilcoxon signed-rank test.

We calculated distance matrices using two phylogeny-independent (Euclidean and Jaccard distances; Jaccard, 1912) and phylogeny-informed (weighted and unweighted UniFrac distances; Lozupone & Knight, 2005) beta diversity distance measures. Notably, to obtain Euclidean distances that satisfy the compositionality constraint of amplicon sequence data (Luz Calle, 2019), we applied a centered-log ratio transformation to unrarefied counts using the *microbiome* package v1.10.0 (Lahti & Sudarshan, 2017). Patterns of beta diversity were visualized using ordinations, and to test for the influence of Sample ID and storage temperature on diversity we ran Permutational Multivariate Analyses of Variance (PERMANOVA) including both effects using the *adonis2* function from *vegan* on the four aforementioned distance matrices at the level of ASVs. The *betadisper* function from *vegan* was also used to test whether the variance in compositional abundances differed between storage temperatures.

## Results

### Community composition

**Figure 1:**
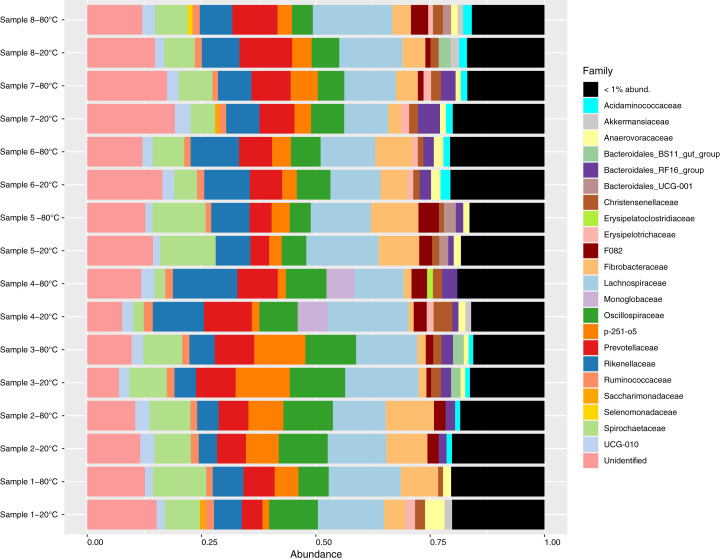
Relative abundance of bacterial families identified in fecal samples collected from feral horses on Sable Island, Nova Scotia, Canada, using 16S rRNA sequencing. Samples from eight horses were divided into aliquots, stored at both −20 °C and −80 °C for 4 years, and characterized using 16S amplicon sequencing. Taxonomy was assigned to amplicon sequence variants (ASVs), and abundances were converted to proportional values then aggregated to the family level. Families with low abundances within a sample (<1%) were pooled for clarity.

Sequencing generated a total of 1,615,043 reads, with a mean of 100,940 reads per sample (87,049–124,423). After sequence processing including quality filtering, denoising, merging and chimera removal, an average of 52,600 reads remained per sample (45,827–64,386). A total of 4427 ASVs were identified, of which 126 were singletons and a further 33 were identified as contaminants. After removing singletons and contaminants, an average of 71.0%, 88.0% and 97.7% of sequences within a sample were assigned to the genus, family and phylum levels with at least 80% confidence, respectively. Bacterial communities were dominated by the *Firmicutes* (average proportion 40.8%; 32.1–53.2%) and *Bacteroidota* phyla (average proportion 35.5%; 22.3–41.4%, Fig. S1). A total of 89 unique families were observed, and 65 of these were present at proportional abundances of 1% or less within a sample (Fig. 1). At the genus level, 138 unique genera were identified of which 114 were present at proportional abundances of 1% or less within a sample (Fig. S2).

### Alpha diversity comparisons

**Figure 2:**
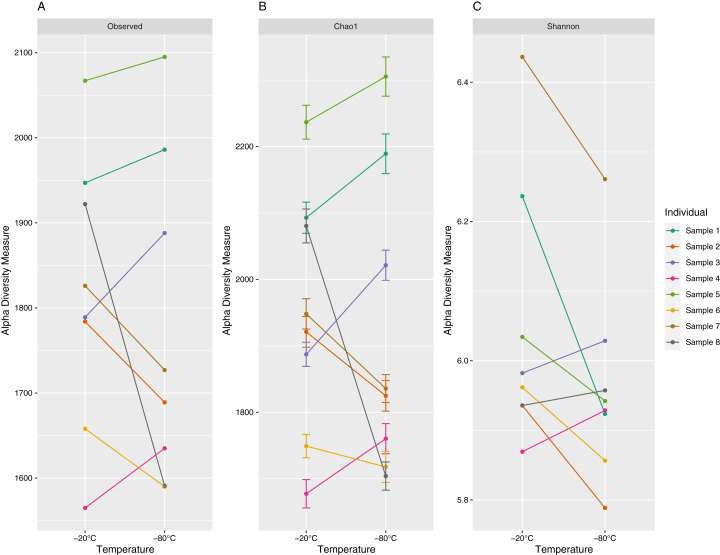
Alpha diversity (richness (A), Choa1 (B) and Shannon indices (C)) of 16S V3–V4 Amplicon Sequence Variants (ASV) for paired equine fecal samples (aliquots) stored at −20 °C and −80 °C for 4 years. Samples were collected from separate individuals on Sable Island, Nova Scotia, Canada. Paired samples (aliquots) are denoted by color.

The number of unique ASVs per sample ranged from 1,565 to 2,095 with a mean (±1 standard deviation) of 1,797 ± 172 (Fig. 2). Chao1 estimates ranged from 1,677 to 2,305 with a mean of 1,935 ± 200, and Shannon values ranged from 5.79 to 6.44 with a mean of 6.00 ± 0.17 (Fig. 2). Observed ASV richness (*p* = 0.39), Chao1 indices (*p* = 0.64) and Shannon diversity (*p* = 0.11) did not differ significantly between samples stored at different temperatures.

### Beta diversity comparisons

**Figure 3:**
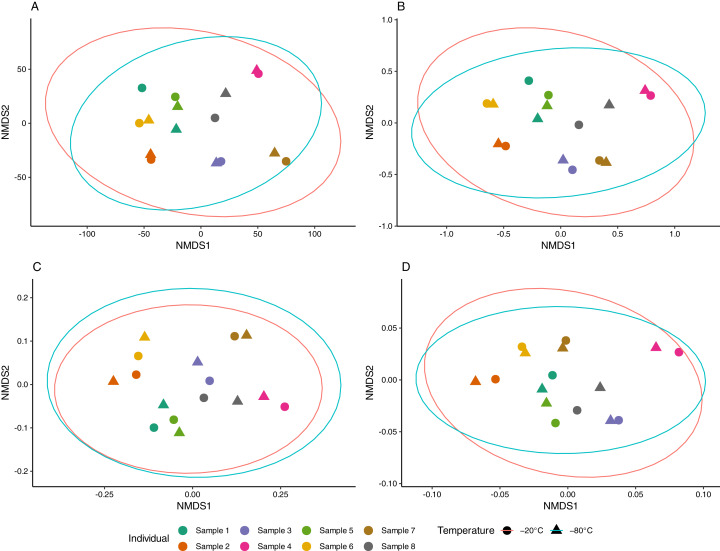
Non-metric multidimensional scaling (NMDS) of equine gut bacterial communities inferred from fecal samples stored at −20 °C and −80 °C. Ordinations were conducted on (A) Euclidean (stress = 0.08), (B) Jaccard (stress = 0.10), (C) Unweighted UniFrac (stress = 0.10) and (D) weighted UniFrac (stress = 0.14) distance matrices from Amplicon Sequencing Variant (ASV) count data. Paired samples share the same color and temperature is denoted by circles (−20 °C) and triangles (−80 °C). Red and blue ellipses represent 95% confidence intervals using the *t*-distributions for samples stored at −20 °C and −80 °C, respectively.

Aliquots from the same sample generally yielded consistent results, as apparent in NMDS ordinations (Fig. 3). The proportion of multivariate variance explained by sample ID was large (79–88%) and statistically significant (*p* < 0.001) for all distance measures considered (Table S1). In contrast, storage temperature explained a comparatively small (2–3%) and non-significant amount of multivariate variation across distances. Similarly, no difference in beta dispersion was observed between temperature storage groups indicating homogeneity of variance in the community structure between treatment groups (Table S1).

## Discussion

Immediate extraction of DNA for amplicon sequencing experiments is preferable but many study systems and clinical applications require the storage of samples by way of freezing. We aimed to determine if equine fecal samples stored at −20 °C for multiple years could recapitulate the bacterial community structure inferred from samples stored at −80 °C. Samples stored at different temperatures did not differ in ASVs richness, Chao1 indices, or evenness (Shannon). Furthermore, variation in community composition among samples was largely driven by sample (individual) ID with no detectable impact of storage temperature. In short, our study demonstrates that archiving equine feces at either −20 °C or −80 °C for multiple years has negligible impacts of 16S rRNA microbiome profiling.

Our findings mirror those obtained in similar studies that have looked at the impact of storage freezing temperature on microbiome profiling (Table 1). For example, Jenkins et al. (2018) noted that murine fecal samples stored at −20 °C and −80 °C for 33 days did not differ in terms of richness or the Chao1 estimator. Microbiome profiles from spider monkey feces stored at −20 °C for 8 weeks closely resembled those stored at −80 °C in composition and diversity (Hale et al., 2015). Using pyrosequencing reads, Hang et al. (2014) did not observe differences between oropharyngeal swabs at these temperatures using weighted UniFrac distances.

Though we did not compare frozen samples to freshly extracted samples, previous studies have shown that long-term storage at −20 °C generally gives rise to microbiota profiles similar to those that have been immediately extracted (Hale et al., 2015; Kia et al., 2016). In addition, limited differences were observed between fresh fecal samples and those stored at −80 °C in RNAlater for 5 years (Tap et al., 2019). Together with our findings, these suggest that archiving fecal samples for long periods of time at −20 °C likely yields uncompromised microbiome profiles, even when compared to DNA extracted from fresh samples. Studies comparing results from fresh fecal samples with those of samples archived at −20 °C for multiple aliquots would be valuable.

Based on this study and other accumulating evidence, we suggest the use of −20 °C as a cost-effective storage temperature to maintain microbiome integrity over multiple years comparable to that of samples stored at −80 °C, at least for targeted sequencing of short amplicons. However, it remains to be seen if this is generalizable to longer time periods such as decades, other sample types or host organisms, or non-amplicon based sequencing methods such as shotgun metagenomics and third generation sequencing. It is also possible that larger sample sizes would lead to the detection of subtle variation, if present. We also note that our study used manual defrost freezers, and that long-term archival of samples in self-defrosting freezers experiencing frequent temperature fluctuations may yield different outcomes.

In addition to general relevance, our findings are especially pertinent to clinicians and researchers conducting field or community-based studies where access to −80 °C storage is limited or absent. Wider adoption and acceptance of storage at −20 °C for amplicon-based microbiome research should greatly increase total storage space, encourage the re-use of samples, and promote longitudinal sampling (Björk et al., 2019). Increased reliance on −20 °C freezers, at least for storage up to a few years, may also help reduce drop-out rates in microbiome studies by alleviating the logistical burden of rapidly transferring samples to −80 °C (Vandeputte et al., 2017). In terms of standardizing study design, DNA extraction and amplification protocols may be of greater importance for resource allocation, which are likely larger sources of technical variation (Costea et al., 2017; Sinha et al., 2017) compared to long-term storage temperature.

## Conclusion

We demonstrated that technical replicates of equine fecal samples stored at −20 °C and −80 °C for multiple years yield similar bacterial microbiome results in terms of alpha and beta diversity. Our findings add to mounting evidence indicating that standard domestic freezers are both economical and effective for microbiome research.

## Supplemental Information

10.7717/peerj.10837/supp-1 Supplemental Information 1 Raw 16S reads 1 for fecal sample SI_2014_E4 stored at −20 °C for 4 years.

10.7717/peerj.10837/supp-2 Supplemental Information 2 Raw 16S reads 2 for fecal sample SI_2014_E4 stored at −20 °C for 4 years.

10.7717/peerj.10837/supp-3 Supplemental Information 3 Raw 16S reads 1 for fecal sample SI_2014_E4 stored at −80 °C for 4 years.

10.7717/peerj.10837/supp-4 Supplemental Information 4 Raw 16S reads 2 for fecal sample SI_2014_E4 stored at −80 °C for 4 years.

10.7717/peerj.10837/supp-5 Supplemental Information 5 Raw 16S reads 1 for fecal sample SI_2014_J83 stored at −20 °C for 4 years.

10.7717/peerj.10837/supp-6 Supplemental Information 6 Raw 16S reads 2 for fecal sample SI_2014_J83 stored at −20 °C for 4 years.

10.7717/peerj.10837/supp-7 Supplemental Information 7 Raw 16S reads 1 for fecal sample SI_2014_J83 stored at −80 °C for 4 years.

10.7717/peerj.10837/supp-8 Supplemental Information 8 Raw 16S reads 2 for fecal sample SI_2014_J83 stored at −80 °C for 4 years.

10.7717/peerj.10837/supp-9 Supplemental Information 9 Raw 16S reads 1 for fecal sample SI_2014_K10 stored at −20 °C for 4 years.

10.7717/peerj.10837/supp-10 Supplemental Information 10 Raw 16S reads 2 for fecal sample SI_2014_K10 stored at −20 °C for 4 years.

10.7717/peerj.10837/supp-11 Supplemental Information 11 Raw 16S reads 1 for fecal sample SI_2014_K10 stored at −80 °C for 4 years.

10.7717/peerj.10837/supp-12 Supplemental Information 12 Raw 16S reads 2 for fecal sample SI_2014_K10 stored at −80 °C for 4 years.

10.7717/peerj.10837/supp-13 Supplemental Information 13 Raw 16S reads 2 for fecal sample SI_2014_K76 stored at −80 °C for 4 years.

10.7717/peerj.10837/supp-14 Supplemental Information 14 Raw 16S reads 1 for fecal sample SI_2014_K76 stored at −80 °C for 4 years.

10.7717/peerj.10837/supp-15 Supplemental Information 15 Raw 16S reads 2 for fecal sample SI_2014_K76 stored at −20 °C for 4 years.

10.7717/peerj.10837/supp-16 Supplemental Information 16 Raw 16S reads 2 for fecal sample SI_2014_K80 stored at −20 °C for 4 years.

10.7717/peerj.10837/supp-17 Supplemental Information 17 Raw 16S reads 2 for fecal sample SI_2014_K80 stored at −80 °C for 4 years.

10.7717/peerj.10837/supp-18 Supplemental Information 18 Raw 16S reads 2 for fecal sample SI_2014_K80 stored at −80 °C for 4 years.

10.7717/peerj.10837/supp-19 Supplemental Information 19 Raw 16S reads 2 for fecal sample SI_2014_K80 stored at −20 °C for 4 years.

10.7717/peerj.10837/supp-20 Supplemental Information 20 Raw 16S reads 2 for fecal sample SI_2014_L06 stored at −20 °C for 4 years.

10.7717/peerj.10837/supp-21 Supplemental Information 21 Raw 16S reads 2 for fecal sample SI_2014_L06 stored at −20 °C for 4 years.

10.7717/peerj.10837/supp-22 Supplemental Information 22 Raw 16S reads 2 for fecal sample SI_2014_L06 stored at −80 °C for 4 years.

10.7717/peerj.10837/supp-23 Supplemental Information 23 Raw 16S reads 2 for fecal sample SI_2014_L06 stored at −80 °C for 4 years.

10.7717/peerj.10837/supp-24 Supplemental Information 24 Raw 16S reads 2 for fecal sample SI_2014_L71 stored at −80 °C for 4 years.

10.7717/peerj.10837/supp-25 Supplemental Information 25 Raw 16S reads 2 for fecal sample SI_2014_L71 stored at −20 °C for 4 years.

10.7717/peerj.10837/supp-26 Supplemental Information 26 Raw 16S reads 2 for fecal sample SI_2014_L71 stored at −20 °C for 4 years.

10.7717/peerj.10837/supp-27 Supplemental Information 27 Raw 16S reads 2 for fecal sample SI_2014_L71 stored at −80 °C for 4 years.

10.7717/peerj.10837/supp-28 Supplemental Information 28 Raw 16S reads 2 for fecal sample SI_2014_S146 stored at −80 °C for 4 years.

10.7717/peerj.10837/supp-29 Supplemental Information 29 Raw 16S reads 2 for fecal sample SI_2014_S146 stored at −20 °C for 4 years.

10.7717/peerj.10837/supp-30 Supplemental Information 30 Raw 16S reads 1 for fecal sample SI_2014_S146 stored at −20 °C for 4 years.

10.7717/peerj.10837/supp-31 Supplemental Information 31 Raw 16S reads 1 for fecal sample SI_2014_S146 stored at −80 °C for 4 years.

10.7717/peerj.10837/supp-32 Supplemental Information 32 Raw 16S reads 1 for Negative Control 1.

10.7717/peerj.10837/supp-33 Supplemental Information 33 Raw 16S reads 2 for Negative Control 1.

10.7717/peerj.10837/supp-34 Supplemental Information 34 Raw 16S reads 1 for Negative Control 2.

10.7717/peerj.10837/supp-35 Supplemental Information 35 Raw 16S reads 2 for Negative Control 2.

10.7717/peerj.10837/supp-36 Supplemental Information 36 Raw 16S reads 1 for No Template Control.

10.7717/peerj.10837/supp-37 Supplemental Information 37 Raw 16S reads 2 for No Template Control.

10.7717/peerj.10837/supp-38 Supplemental Information 38 Information about samples included in the study.

10.7717/peerj.10837/supp-39 Supplemental Information 39 Summary statistics for the comparison of equine fecal samples stored at −20 °C and −80 °C using 16S amplicon-sequencing. *R*^2^ and *p* values from permutational multivariate analysis of variance (PERMANOVA) conducted to test for differences between temperature treatments are presented for the four measures of dissimilarity considered (9999 permutations).

10.7717/peerj.10837/supp-40 Supplemental Information 40 Relative abundance of bacterial phyla identified in fecal samples collected from feral horses on Sable Island, Nova Scotia, Canada, using 16S rRNA sequencing. Samples from eight horses were divided into aliquots, stored at both −20 °C and −80 °C for 4 years, and characterized using 16S amplicon sequencing. Taxonomy was assigned to amplicon sequence variants (ASVs), and abundances were converted to proportional values then aggregated to the phylum level.

10.7717/peerj.10837/supp-41 Supplemental Information 41 Relative abundance of bacterial genus identified in fecal samples collected from feral horses on Sable Island, Nova Scotia, Canada, using 16S rRNA sequencing. Samples from eight horses were divided into aliquots, stored at both −20 °C and −80 °C for 4 years, and characterized using 16S amplicon sequencing. Taxonomy was assigned to amplicon sequence variants (ASVs), and abundances were converted to proportional values then aggregated to the genus level. Genus with low abundances within a sample (<1%) were pooled for clarity.

10.7717/peerj.10837/supp-42 Supplemental Information 42 Pipeline to analyze 16S data.

10.7717/peerj.10837/supp-43 Supplemental Information 43 R script to analyze processed ASVs.

## Additional Information and Declarations

### Field Study Permissions

The following information was supplied relating to field study approvals (i.e., approving body and any reference numbers): Sample collection and subsequent laboratory analyses were performed under Parks Canada Agency Research and Collections Permit SINP-2013-2014.

### Data Availability

The following information was supplied regarding data availability: Raw 16S sequences are available at NCBI, BioProject: PRJNA674675 (SRX9507884–SRX9507891, SRX9440373, SRX9440395, SRX9440405, SRX9440422, SRX9440423, SRX9440428, SRX9440439, SRX9440443, SRX9517110, SRX9517111, SRX9732812). Raw sequences and code are available as Supplemental Files.
