# Supplementary material for: Long-term storage of feces at −80 °C versus −20 °C is negligible for 16S rRNA amplicon profiling of the equine bacterial microbiome
Source: PeerJ. 2021 Mar 9;9:e10837. doi: 10.7717/peerj.10837 (PMC7953882; doi:10.7717/peerj.10837)
Supplement: Supplemental Information 39 — R2 and p values from permutational multivariate analysis of variance (PERMANOVA) conducted to test for differences between temperature treatments are presented for the four measures of dissimilarity considered (9999 permutations). [file peerj-09-10837-s039.doc]

**Supplementary Table 1: Summary statistics for the comparison of equine fecal samples stored at -20°C and -80°C using 16S amplicon-sequencing.** R^2^ and p values from permutational multivariate analysis of variance (PERMANOVA) conducted to test for differences between temperature treatments are presented for the four measures of dissimilarity considered (9999 permutations).

| **Dissimilarity measure** | **Factor** | ***R^2^*** | ***p (PERMANOVA)*** | ***p (Beta dispersion)*** |
| --- | --- | --- | --- | --- |
| **Euclidean** | Sample ID | 0.84337 | 0.0001 | 0.5254 |
|  | Temperature | 0.02009 | 0.4634 |  |
| **Jaccard** | Sample ID | 0.84399 | 0.0001 | 0.5687 |
|  | Temperature | 0.02092 | 0.3975 |  |
| **Unweighted UniFrac** | Sample ID | 0.79371 | 0.0001 | 0.6124 |
|  | Temperature | 0.03010 | 0.2540 |  |
| **Weighted UniFrac** | Sample ID | 0.87618 | 0.0001 | 0.6829 |
|  | Temperature | 0.01859 | 0.2751 |  |
