# Supplementary material for: Long-term storage of feces at −80 °C versus −20 °C is negligible for 16S rRNA amplicon profiling of the equine bacterial microbiome
Source: PeerJ. 2021 Mar 9;9:e10837. doi: 10.7717/peerj.10837 (PMC7953882; doi:10.7717/peerj.10837)
Supplement: Supplemental Information 41 — Samples from eight horses were divided into aliquots, stored at both −20 °C and −80 °C for 4 years, and characterized using 16S amplicon sequencing. Taxonomy was assigned to amplicon sequence variants (ASVs), and abundances were converted to proportional values then aggregated to the genus level. Genus with low abundances within a sample (<1%) were pooled for clarity. [file peerj-09-10837-s041.pdf]

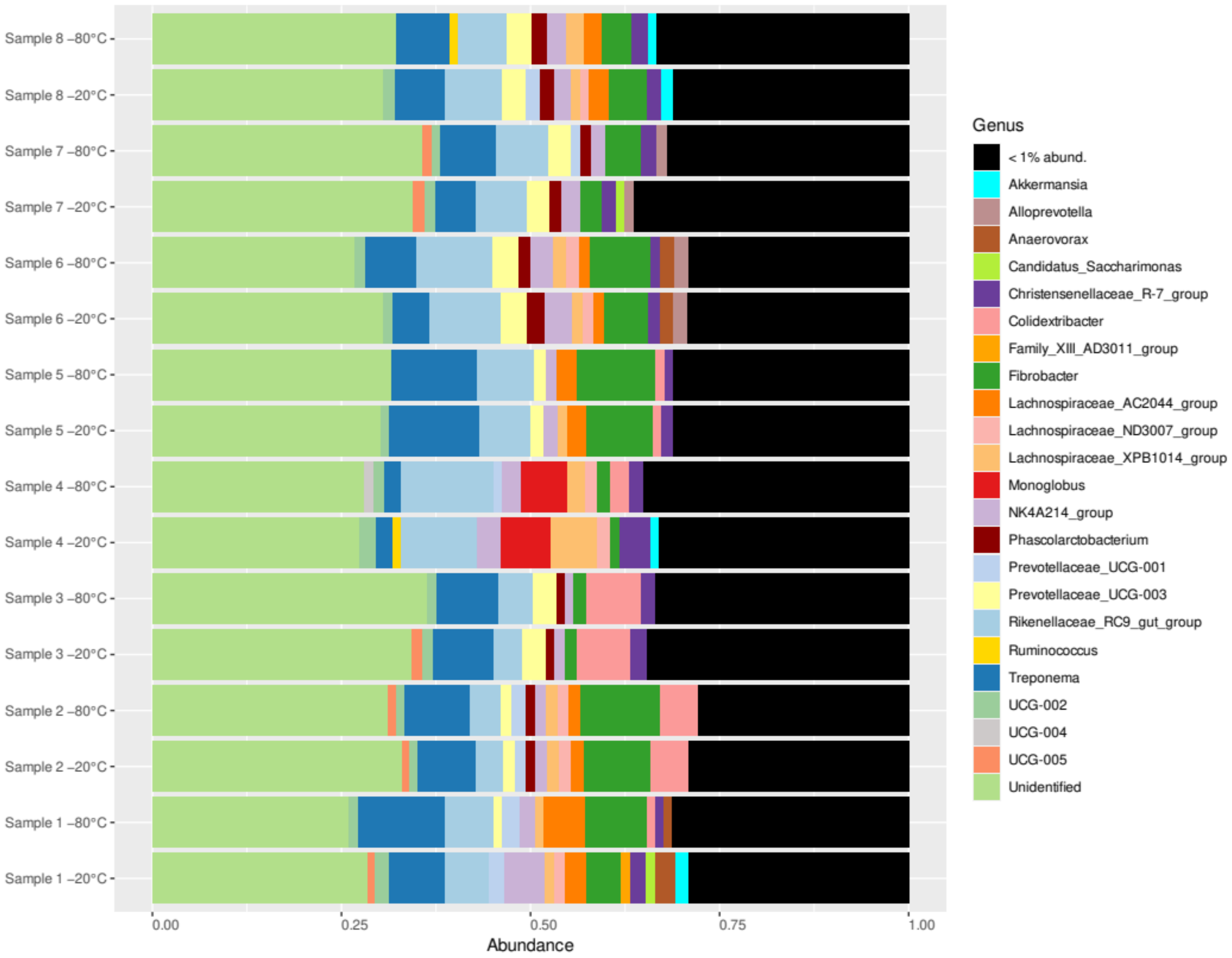

Supplementary Figure 1: Abundances of bacterial genera identified in fecal samples collected from feral horses on Sable Island, Nova Scotia, Canada, using 16S rRNA sequencing. Samples from eight horses were divided into aliquots, stored at both -20°C and -80°C for four years, and characterized using 16S amplicon sequencing. Taxonomy was assigned to amplicon sequence variants (ASVs), and abundances were made proportional then aggregated to the family level. Genera with low abundances (< 1% within a sample) were pooled for clarity.
